# Supplementary material for: Estimating Recent and Historical Effective Population Size of Marine and Freshwater Sticklebacks
Source: Mol Ecol. 2025 Jun 8;34(13):e17825. doi: 10.1111/mec.17825 (PMC12186715; doi:10.1111/mec.17825)
Supplement: Supplementary file 1 — Data S1. [file MEC-34-e17825-s001.pdf]

Supplemental Information for manuscript:

Estimating recent and historical effective population size of marine and freshwater sticklebacks

Xueyun Feng[1,2], Ari Löytynoja[2], Juha Merilä[1,3]

[1]Organismal and Evolutionary Biology Programme, University of Helsinki, FI-00014 University of Helsinki, Finland

[2]Institute of Biotechnology, University of Helsinki, FI-00014 University of Helsinki, Finland

[3]Area of Ecology and Biodiversity, School of Biological Sciences, University of Hong Kong, Hong Kong SAR

List of Figures

S1. Map illustrating sampling locations
S2. Linkage disequilibrium (LD) decay curve in each population
S3. Relatedness (r_{xy}) for pairs of individuals within each population
S4. Effective population size (N_e) of each population estimated with GONE
S5. Estimates of contemporary effective population size (N_e^C) for 45 nine-spined stickleback populations
S6. Historical effective population sizes (N_e) for each population inferred with MSMC2
S7. Historical effective population sizes (N_e) for populations within different ecotypes inferred with MSMC2
S8. Temporal reconstruction of effective population size (N_e) for 45 nine-spined stickleback populations over the past 30,000 years

List of Tables

S1. Sample information
S2. Data filtering for each analysis
S4. Number of SNPs used in GONE analysis
S4. Number of SNPs used in CurrentNe2 analysis
S5. Estimates of contemporary effective population size (N_e^C) using CurrentNe2 and GONE

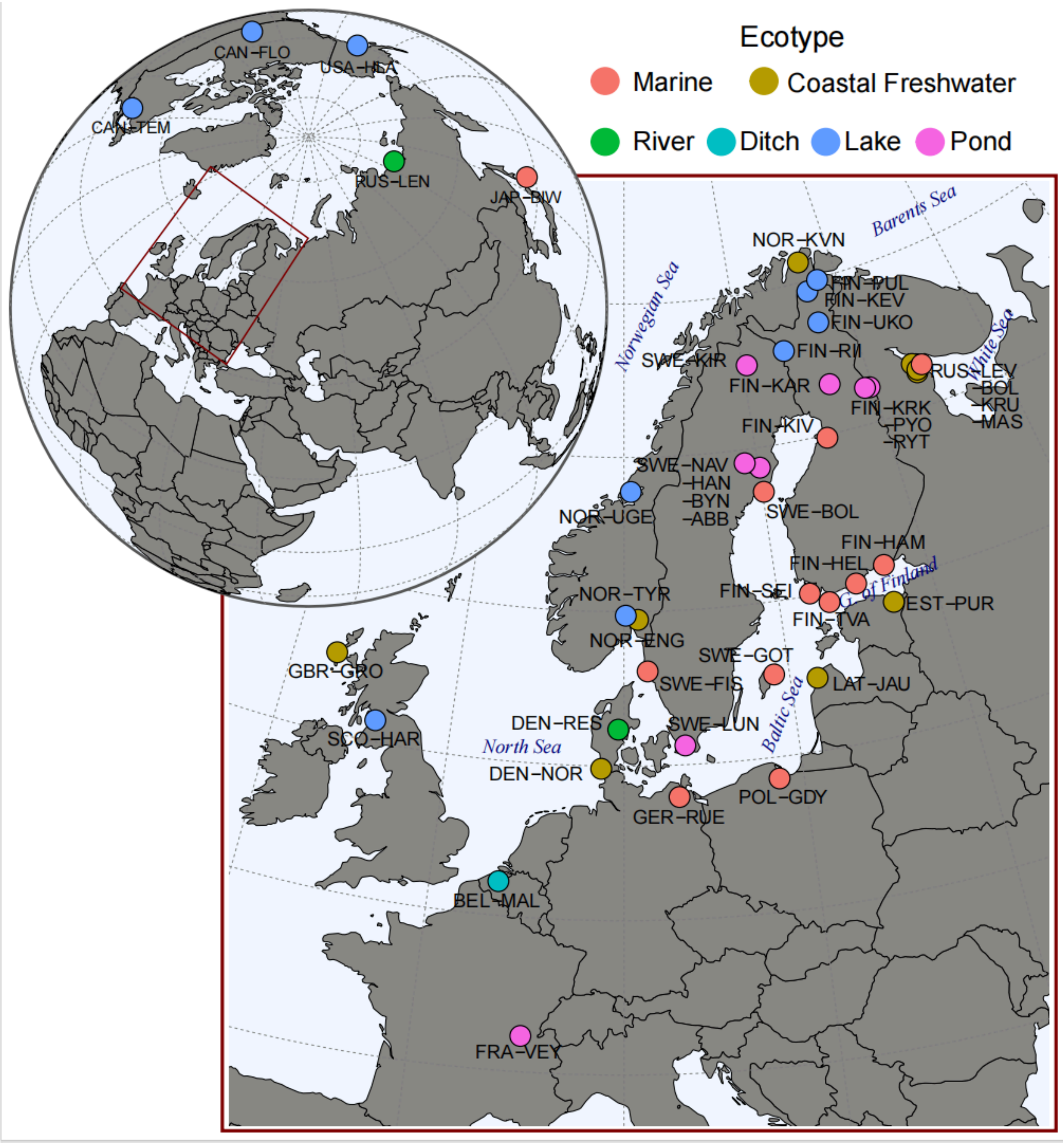

Figure S1. Map illustrating sampling locations of the studied populations, derived from Feng et al., (2022). The ecotypes are indicated by the colors on top.

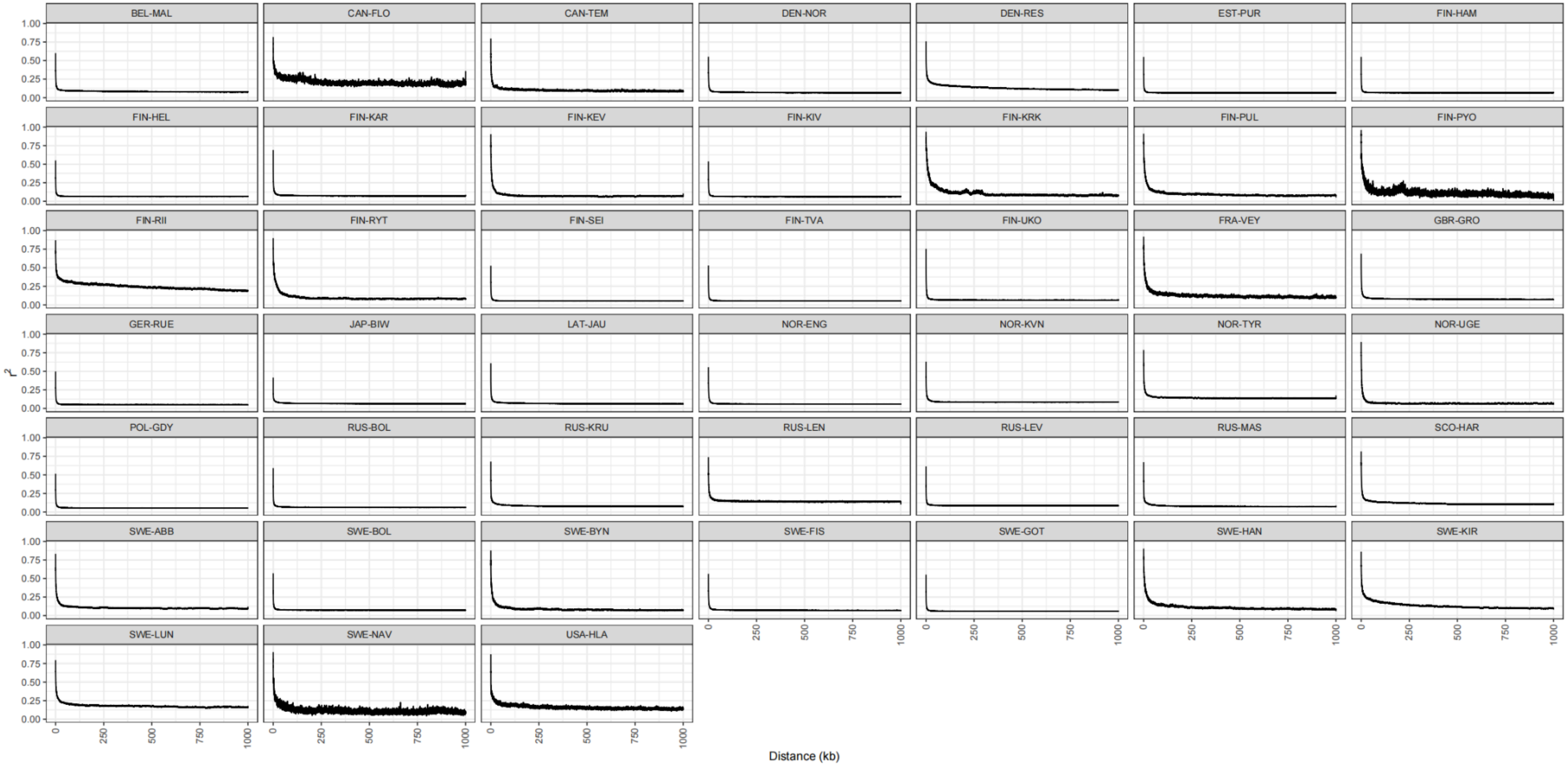

Figure S2. Linkage disequilibrium (LD) decay curve in each population.

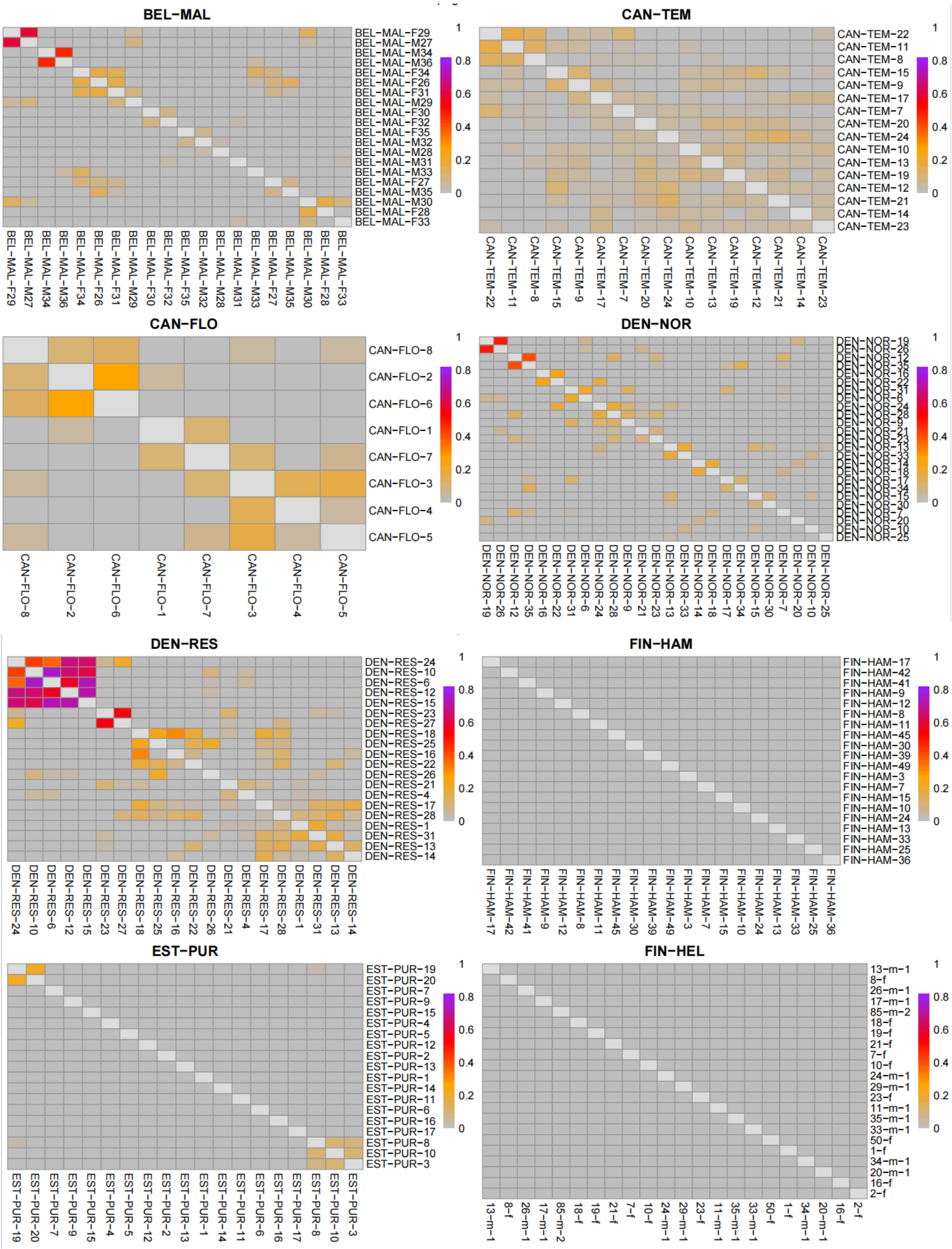

BEL-MAL
BEL-MAL-F29
BEL-MAL-M27
BEL-MAL-M34
BEL-MAL-M36
BEL-MAL-F34
BEL-MAL-F26
BEL-MAL-F31
BEL-MAL-M29
BEL-MAL-F30
BEL-MAL-F32
BEL-MAL-F35
BEL-MAL-M32
BEL-MAL-M28
BEL-MAL-M31
BEL-MAL-M33
BEL-MAL-F27
BEL-MAL-M35
BEL-MAL-M30
BEL-MAL-F28
BEL-MAL-F33
1
0.8
0.6
0.4
0.2
0
CAN-TEM
CAN-TEM-22
CAN-TEM-11
CAN-TEM-8
CAN-TEM-15
CAN-TEM-9
CAN-TEM-17
CAN-TEM-7
CAN-TEM-20
CAN-TEM-24
CAN-TEM-10
CAN-TEM-13
CAN-TEM-19
CAN-TEM-12
CAN-TEM-21
CAN-TEM-14
CAN-TEM-23
CAN-FLO
CAN-FLO-8
CAN-FLO-2
CAN-FLO-6
CAN-FLO-1
CAN-FLO-7
CAN-FLO-3
CAN-FLO-4
CAN-FLO-5
DEN-NOR
DEN-NOR-19
DEN-NOR-26
DEN-NOR-12
DEN-NOR-35
DEN-NOR-16
DEN-NOR-22
DEN-NOR-31
DEN-NOR-6
DEN-NOR-24
DEN-NOR-28
DEN-NOR-9
DEN-NOR-21
DEN-NOR-23
DEN-NOR-13
DEN-NOR-33
DEN-NOR-14
DEN-NOR-18
DEN-NOR-17
DEN-NOR-34
DEN-NOR-15
DEN-NOR-30
DEN-NOR-7
DEN-NOR-20
DEN-NOR-10
DEN-NOR-25
DEN-RES
DEN-RES-24
DEN-RES-10
DEN-RES-6
DEN-RES-12
DEN-RES-15
DEN-RES-23
DEN-RES-27
DEN-RES-18
DEN-RES-25
DEN-RES-16
DEN-RES-22
DEN-RES-26
DEN-RES-21
DEN-RES-4
DEN-RES-17
DEN-RES-28
DEN-RES-1
DEN-RES-31
DEN-RES-13
DEN-RES-14
FIN-HAM
FIN-HAM-17
FIN-HAM-42
FIN-HAM-41
FIN-HAM-9
FIN-HAM-12
FIN-HAM-8
FIN-HAM-11
FIN-HAM-45
FIN-HAM-30
FIN-HAM-39
FIN-HAM-49
FIN-HAM-3
FIN-HAM-7
FIN-HAM-15
FIN-HAM-10
FIN-HAM-24
FIN-HAM-13
FIN-HAM-33
FIN-HAM-25
FIN-HAM-36
EST-PUR
EST-PUR-19
EST-PUR-20
EST-PUR-7
EST-PUR-9
EST-PUR-15
EST-PUR-4
EST-PUR-5
EST-PUR-12
EST-PUR-2
EST-PUR-13
EST-PUR-1
EST-PUR-14
EST-PUR-11
EST-PUR-6
EST-PUR-16
EST-PUR-17
EST-PUR-8
EST-PUR-10
EST-PUR-3
FIN-HEL
13-m-1
8-f
26-m-1
17-m-1
85-m-2
18-f
19-f
21-f
7-f
10-f
24-m-1
29-m-1
23-f
11-m-1
35-m-1
33-m-1
50-f
1-f
34-m-1
20-m-1
16-f
2-f

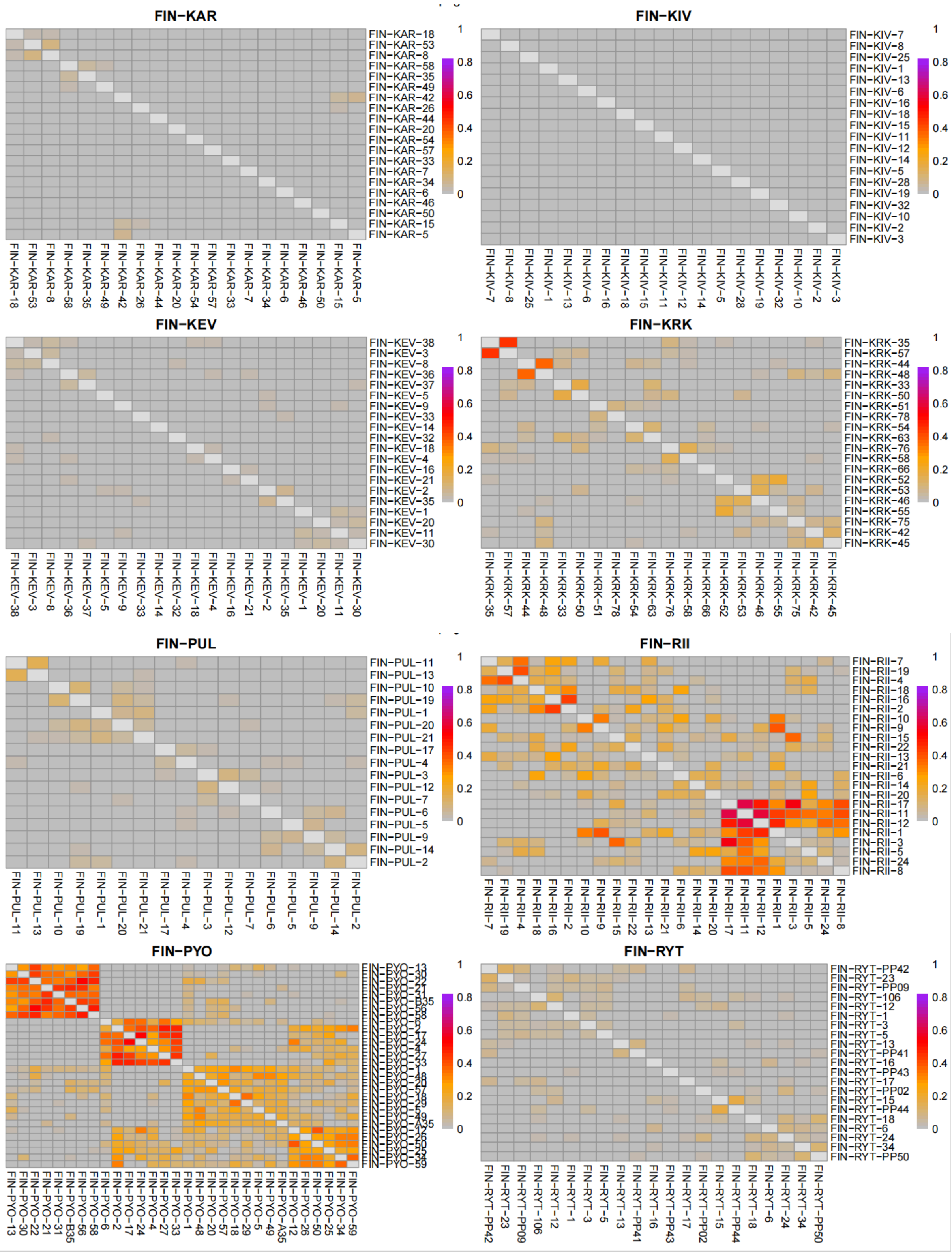
FIN−KAR
FIN−KAR−18 FIN−KAR−53 FIN−KAR−8 FIN−KAR−58 FIN−KAR−35 FIN−KAR−49 FIN−KAR−42 FIN−KAR−26 FIN−KAR−44 FIN−KAR−20 FIN−KAR−54 FIN−KAR−57 FIN−KAR−33 FIN−KAR−7 FIN−KAR−34 FIN−KAR−6 FIN−KAR−46 FIN−KAR−50 FIN−KAR−15 FIN−KAR−5
FIN−KIV
FIN−KIV−7 FIN−KIV−8 FIN−KIV−25 FIN−KIV−1 FIN−KIV−13 FIN−KIV−6 FIN−KIV−16 FIN−KIV−18 FIN−KIV−15 FIN−KIV−11 FIN−KIV−12 FIN−KIV−14 FIN−KIV−5 FIN−KIV−28 FIN−KIV−19 FIN−KIV−32 FIN−KIV−10 FIN−KIV−2 FIN−KIV−3
FIN−KEV
FIN−KEV−38 FIN−KEV−3 FIN−KEV−8 FIN−KEV−36 FIN−KEV−37 FIN−KEV−5 FIN−KEV−9 FIN−KEV−33 FIN−KEV−14 FIN−KEV−32 FIN−KEV−18 FIN−KEV−4 FIN−KEV−16 FIN−KEV−21 FIN−KEV−2 FIN−KEV−35 FIN−KEV−1 FIN−KEV−20 FIN−KEV−11 FIN−KEV−30
FIN−KRK
FIN−KRK−35 FIN−KRK−57 FIN−KRK−44 FIN−KRK−48 FIN−KRK−33 FIN−KRK−50 FIN−KRK−51 FIN−KRK−78 FIN−KRK−54 FIN−KRK−63 FIN−KRK−76 FIN−KRK−58 FIN−KRK−66 FIN−KRK−52 FIN−KRK−53 FIN−KRK−46 FIN−KRK−55 FIN−KRK−75 FIN−KRK−42 FIN−KRK−45
FIN−PUL
FIN−PUL−11 FIN−PUL−13 FIN−PUL−10 FIN−PUL−19 FIN−PUL−1 FIN−PUL−20 FIN−PUL−21 FIN−PUL−17 FIN−PUL−4 FIN−PUL−3 FIN−PUL−12 FIN−PUL−7 FIN−PUL−6 FIN−PUL−5 FIN−PUL−9 FIN−PUL−14 FIN−PUL−2
FIN−RII
FIN−RII−7 FIN−RII−19 FIN−RII−4 FIN−RII−18 FIN−RII−16 FIN−RII−2 FIN−RII−10 FIN−RII−9 FIN−RII−15 FIN−RII−22 FIN−RII−13 FIN−RII−21 FIN−RII−6 FIN−RII−14 FIN−RII−20 FIN−RII−17 FIN−RII−11 FIN−RII−12 FIN−RII−1 FIN−RII−3 FIN−RII−5 FIN−RII−24 FIN−RII−8
FIN−PYO
FIN−PYO−13 FIN−PYO−30 FIN−PYO−22 FIN−PYO−21 FIN−PYO−31 FIN−PYO−B35 FIN−PYO−56 FIN−PYO−58 FIN−PYO−6 FIN−PYO−2 FIN−PYO−17 FIN−PYO−24 FIN−PYO−4 FIN−PYO−27 FIN−PYO−33 FIN−PYO−1 FIN−PYO−48 FIN−PYO−20 FIN−PYO−57 FIN−PYO−18 FIN−PYO−29 FIN−PYO−5 FIN−PYO−49 FIN−PYO−A35 FIN−PYO−12 FIN−PYO−26 FIN−PYO−50 FIN−PYO−25 FIN−PYO−34 FIN−PYO−59
FIN−RYT
FIN−RYT−PP42 FIN−RYT−23 FIN−RYT−PP09 FIN−RYT−106 FIN−RYT−12 FIN−RYT−1 FIN−RYT−3 FIN−RYT−5 FIN−RYT−13 FIN−RYT−PP41 FIN−RYT−16 FIN−RYT−PP43 FIN−RYT−17 FIN−RYT−PP02 FIN−RYT−15 FIN−RYT−PP44 FIN−RYT−18 FIN−RYT−6 FIN−RYT−24 FIN−RYT−34 FIN−RYT−PP50
1 0.8 0.6 0.4 0.2 0

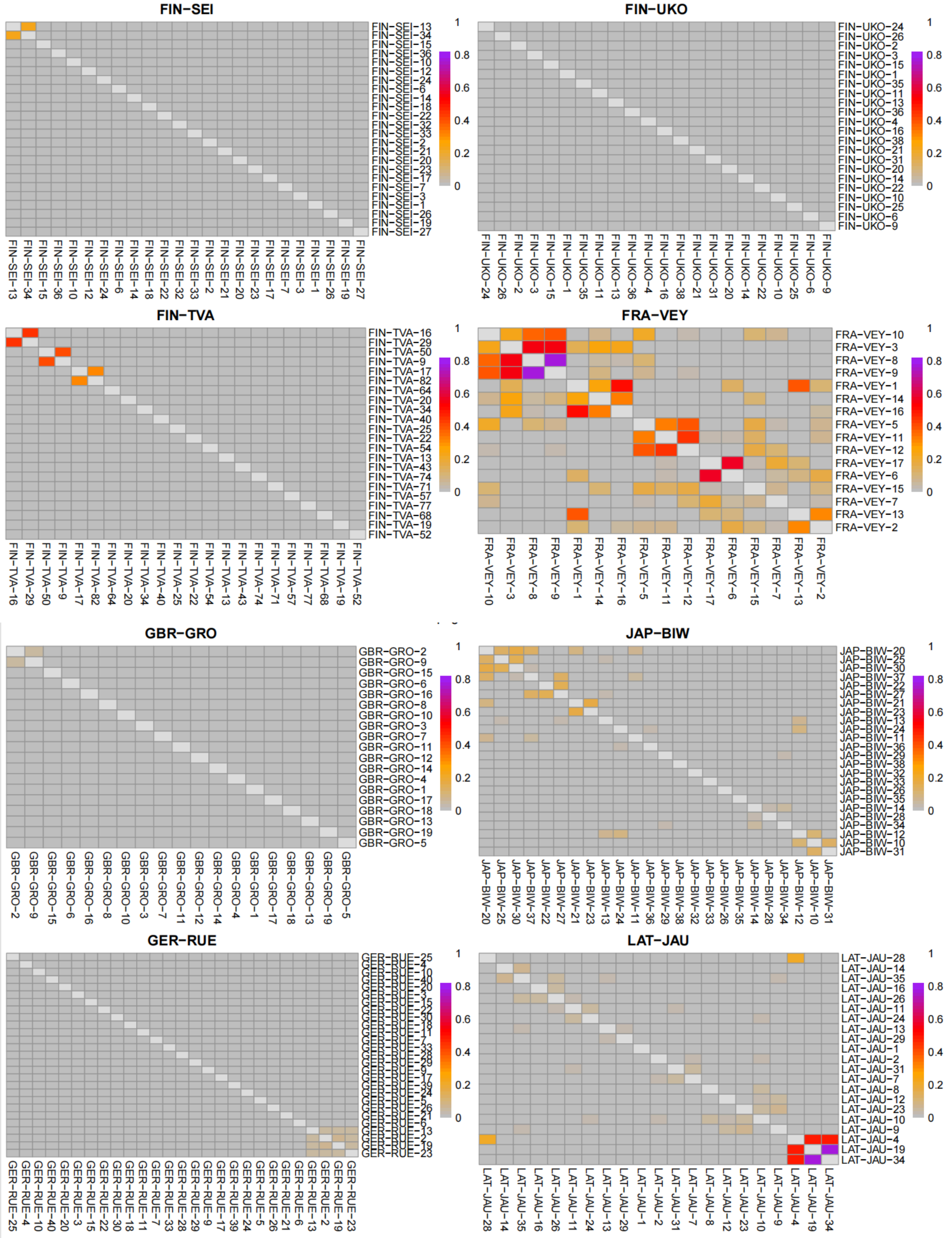
FIN−SEI
FIN−SEI−13 FIN−SEI−34 FIN−SEI−15 FIN−SEI−36 FIN−SEI−10 FIN−SEI−12 FIN−SEI−24 FIN−SEI−6 FIN−SEI−14 FIN−SEI−18 FIN−SEI−22 FIN−SEI−32 FIN−SEI−33 FIN−SEI−2 FIN−SEI−21 FIN−SEI−20 FIN−SEI−23 FIN−SEI−17 FIN−SEI−7 FIN−SEI−3 FIN−SEI−1 FIN−SEI−26 FIN−SEI−19 FIN−SEI−27
FIN−UKO
FIN−UKO−24 FIN−UKO−26 FIN−UKO−2 FIN−UKO−3 FIN−UKO−15 FIN−UKO−1 FIN−UKO−35 FIN−UKO−11 FIN−UKO−13 FIN−UKO−36 FIN−UKO−4 FIN−UKO−16 FIN−UKO−38 FIN−UKO−21 FIN−UKO−31 FIN−UKO−20 FIN−UKO−14 FIN−UKO−22 FIN−UKO−10 FIN−UKO−25 FIN−UKO−6 FIN−UKO−9
FIN−TVA
FIN−TVA−16 FIN−TVA−29 FIN−TVA−50 FIN−TVA−9 FIN−TVA−17 FIN−TVA−82 FIN−TVA−64 FIN−TVA−20 FIN−TVA−34 FIN−TVA−40 FIN−TVA−25 FIN−TVA−22 FIN−TVA−54 FIN−TVA−13 FIN−TVA−43 FIN−TVA−74 FIN−TVA−71 FIN−TVA−57 FIN−TVA−77 FIN−TVA−68 FIN−TVA−19 FIN−TVA−52
FRA−VEY
FRA−VEY−10 FRA−VEY−3 FRA−VEY−8 FRA−VEY−9 FRA−VEY−1 FRA−VEY−14 FRA−VEY−16 FRA−VEY−5 FRA−VEY−11 FRA−VEY−12 FRA−VEY−17 FRA−VEY−6 FRA−VEY−15 FRA−VEY−7 FRA−VEY−13 FRA−VEY−2
GBR−GRO
GBR−GRO−2 GBR−GRO−9 GBR−GRO−15 GBR−GRO−6 GBR−GRO−16 GBR−GRO−8 GBR−GRO−10 GBR−GRO−3 GBR−GRO−7 GBR−GRO−11 GBR−GRO−12 GBR−GRO−14 GBR−GRO−4 GBR−GRO−1 GBR−GRO−17 GBR−GRO−18 GBR−GRO−13 GBR−GRO−19 GBR−GRO−5
JAP−BIW
JAP−BIW−20 JAP−BIW−25 JAP−BIW−30 JAP−BIW−37 JAP−BIW−22 JAP−BIW−27 JAP−BIW−21 JAP−BIW−23 JAP−BIW−13 JAP−BIW−24 JAP−BIW−11 JAP−BIW−36 JAP−BIW−29 JAP−BIW−38 JAP−BIW−32 JAP−BIW−33 JAP−BIW−26 JAP−BIW−35 JAP−BIW−14 JAP−BIW−28 JAP−BIW−34 JAP−BIW−12 JAP−BIW−10 JAP−BIW−31
GER−RUE
GER−RUE−25 GER−RUE−4 GER−RUE−10 GER−RUE−40 GER−RUE−20 GER−RUE−3 GER−RUE−15 GER−RUE−22 GER−RUE−30 GER−RUE−18 GER−RUE−11 GER−RUE−7 GER−RUE−33 GER−RUE−28 GER−RUE−29 GER−RUE−9 GER−RUE−17 GER−RUE−39 GER−RUE−24 GER−RUE−5 GER−RUE−26 GER−RUE−21 GER−RUE−6 GER−RUE−13 GER−RUE−2 GER−RUE−19 GER−RUE−23
LAT−JAU
LAT−JAU−28 LAT−JAU−14 LAT−JAU−35 LAT−JAU−16 LAT−JAU−26 LAT−JAU−11 LAT−JAU−24 LAT−JAU−13 LAT−JAU−29 LAT−JAU−1 LAT−JAU−2 LAT−JAU−31 LAT−JAU−7 LAT−JAU−8 LAT−JAU−12 LAT−JAU−23 LAT−JAU−10 LAT−JAU−9 LAT−JAU−4 LAT−JAU−19 LAT−JAU−34
1
0.8
0.6
0.4
0.2
0

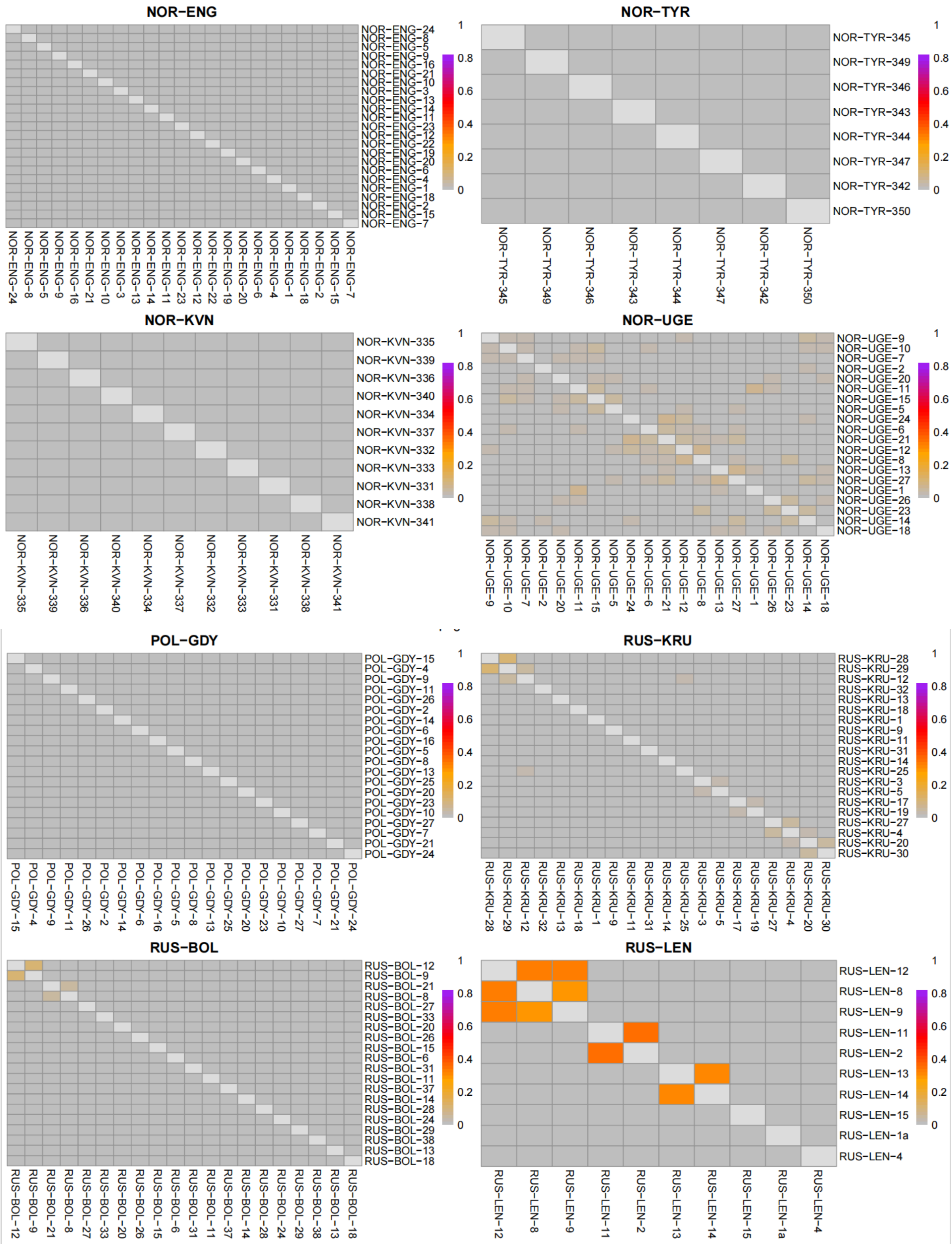

NOR−ENG
NOR−ENG−24 NOR−ENG−8 NOR−ENG−5 NOR−ENG−9 NOR−ENG−16 NOR−ENG−21 NOR−ENG−10 NOR−ENG−3 NOR−ENG−13 NOR−ENG−14 NOR−ENG−11 NOR−ENG−23 NOR−ENG−12 NOR−ENG−22 NOR−ENG−19 NOR−ENG−20 NOR−ENG−6 NOR−ENG−4 NOR−ENG−1 NOR−ENG−18 NOR−ENG−2 NOR−ENG−15 NOR−ENG−7
NOR−TYR
NOR−TYR−345 NOR−TYR−349 NOR−TYR−346 NOR−TYR−343 NOR−TYR−344 NOR−TYR−347 NOR−TYR−342 NOR−TYR−350
NOR−KVN
NOR−KVN−335 NOR−KVN−339 NOR−KVN−336 NOR−KVN−340 NOR−KVN−334 NOR−KVN−337 NOR−KVN−332 NOR−KVN−333 NOR−KVN−331 NOR−KVN−338 NOR−KVN−341
NOR−UGE
NOR−UGE−9 NOR−UGE−10 NOR−UGE−7 NOR−UGE−2 NOR−UGE−20 NOR−UGE−11 NOR−UGE−15 NOR−UGE−5 NOR−UGE−24 NOR−UGE−6 NOR−UGE−21 NOR−UGE−12 NOR−UGE−8 NOR−UGE−13 NOR−UGE−27 NOR−UGE−1 NOR−UGE−26 NOR−UGE−23 NOR−UGE−14 NOR−UGE−18
POL−GDY
POL−GDY−15 POL−GDY−4 POL−GDY−9 POL−GDY−11 POL−GDY−26 POL−GDY−2 POL−GDY−14 POL−GDY−6 POL−GDY−16 POL−GDY−5 POL−GDY−8 POL−GDY−13 POL−GDY−25 POL−GDY−20 POL−GDY−23 POL−GDY−10 POL−GDY−27 POL−GDY−7 POL−GDY−21 POL−GDY−24
RUS−KRU
RUS−KRU−28 RUS−KRU−29 RUS−KRU−12 RUS−KRU−32 RUS−KRU−13 RUS−KRU−18 RUS−KRU−1 RUS−KRU−9 RUS−KRU−11 RUS−KRU−31 RUS−KRU−14 RUS−KRU−25 RUS−KRU−3 RUS−KRU−5 RUS−KRU−17 RUS−KRU−19 RUS−KRU−27 RUS−KRU−4 RUS−KRU−20 RUS−KRU−30
RUS−BOL
RUS−BOL−12 RUS−BOL−9 RUS−BOL−21 RUS−BOL−8 RUS−BOL−27 RUS−BOL−33 RUS−BOL−20 RUS−BOL−26 RUS−BOL−15 RUS−BOL−6 RUS−BOL−31 RUS−BOL−11 RUS−BOL−37 RUS−BOL−14 RUS−BOL−28 RUS−BOL−24 RUS−BOL−29 RUS−BOL−38 RUS−BOL−13 RUS−BOL−18
RUS−LEN
RUS−LEN−12 RUS−LEN−8 RUS−LEN−9 RUS−LEN−11 RUS−LEN−2 RUS−LEN−13 RUS−LEN−14 RUS−LEN−15 RUS−LEN−1a RUS−LEN−4
1 0.8 0.6 0.4 0.2 0

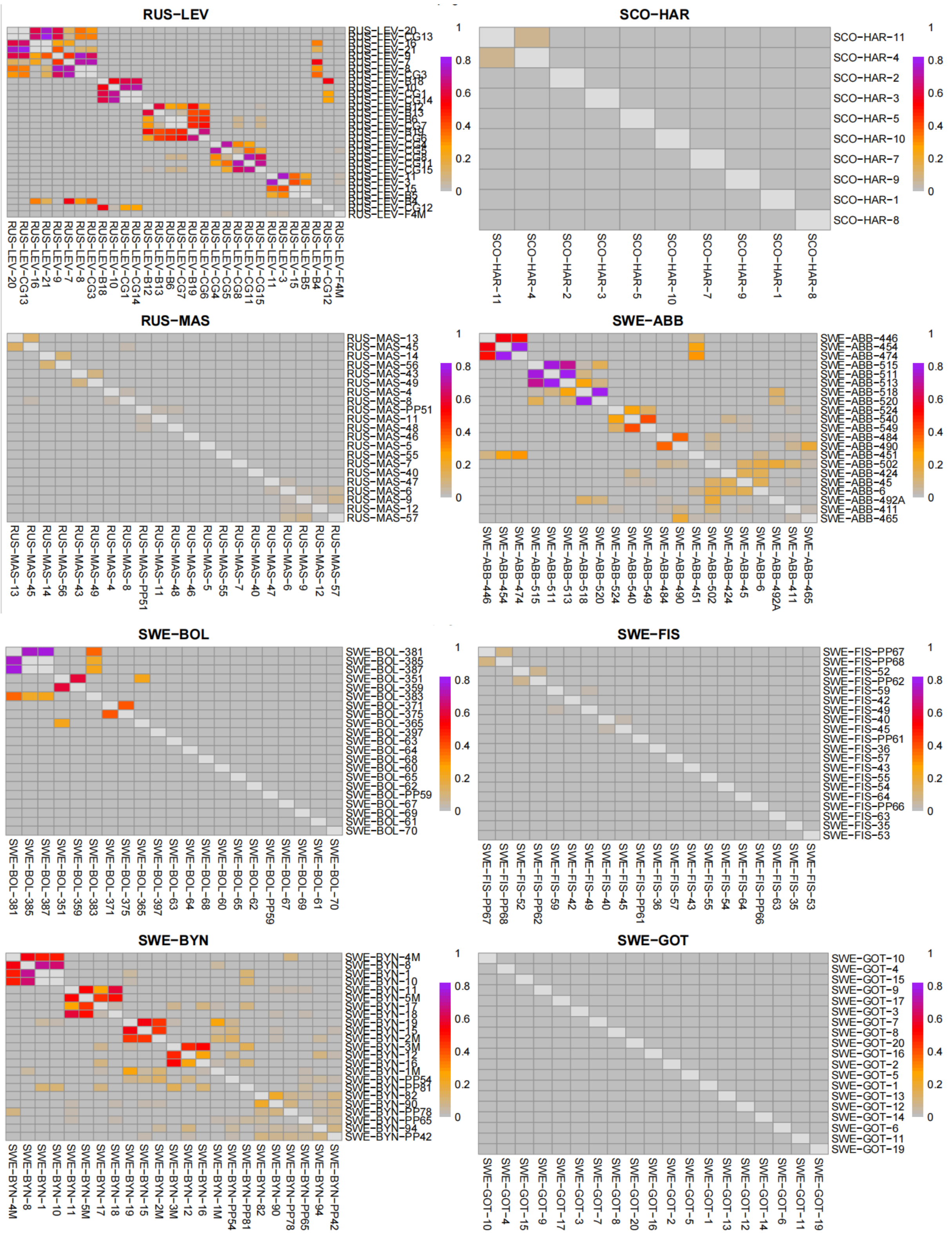
RUS−LEV
RUS−LEV−20 RUS−LEV−CG13 RUS−LEV−16 RUS−LEV−21 RUS−LEV−9 RUS−LEV−7 RUS−LEV−8 RUS−LEV−CG3 RUS−LEV−B18 RUS−LEV−10 RUS−LEV−CG1 RUS−LEV−CG14 RUS−LEV−B12 RUS−LEV−B13 RUS−LEV−B6 RUS−LEV−CG7 RUS−LEV−B19 RUS−LEV−CG6 RUS−LEV−CG4 RUS−LEV−CG8 RUS−LEV−CG5 RUS−LEV−CG11 RUS−LEV−CG15 RUS−LEV−11 RUS−LEV−3 RUS−LEV−15 RUS−LEV−B5 RUS−LEV−B4 RUS−LEV−CG12 RUS−LEV−F4M
SCO−HAR
SCO−HAR−11 SCO−HAR−4 SCO−HAR−2 SCO−HAR−3 SCO−HAR−5 SCO−HAR−10 SCO−HAR−7 SCO−HAR−9 SCO−HAR−1 SCO−HAR−8
RUS−MAS
RUS−MAS−13 RUS−MAS−45 RUS−MAS−14 RUS−MAS−56 RUS−MAS−43 RUS−MAS−49 RUS−MAS−4 RUS−MAS−8 RUS−MAS−PP51 RUS−MAS−11 RUS−MAS−48 RUS−MAS−46 RUS−MAS−5 RUS−MAS−55 RUS−MAS−7 RUS−MAS−40 RUS−MAS−47 RUS−MAS−6 RUS−MAS−9 RUS−MAS−12 RUS−MAS−57
SWE−ABB
SWE−ABB−446 SWE−ABB−454 SWE−ABB−474 SWE−ABB−515 SWE−ABB−511 SWE−ABB−513 SWE−ABB−518 SWE−ABB−520 SWE−ABB−524 SWE−ABB−540 SWE−ABB−549 SWE−ABB−484 SWE−ABB−490 SWE−ABB−451 SWE−ABB−502 SWE−ABB−424 SWE−ABB−45 SWE−ABB−6 SWE−ABB−492A SWE−ABB−411 SWE−ABB−465
SWE−BOL
SWE−BOL−381 SWE−BOL−385 SWE−BOL−387 SWE−BOL−351 SWE−BOL−359 SWE−BOL−383 SWE−BOL−371 SWE−BOL−375 SWE−BOL−365 SWE−BOL−397 SWE−BOL−63 SWE−BOL−64 SWE−BOL−68 SWE−BOL−60 SWE−BOL−65 SWE−BOL−62 SWE−BOL−PP59 SWE−BOL−67 SWE−BOL−69 SWE−BOL−61 SWE−BOL−70
SWE−FIS
SWE−FIS−PP67 SWE−FIS−PP68 SWE−FIS−52 SWE−FIS−PP62 SWE−FIS−59 SWE−FIS−42 SWE−FIS−49 SWE−FIS−40 SWE−FIS−45 SWE−FIS−PP61 SWE−FIS−36 SWE−FIS−57 SWE−FIS−43 SWE−FIS−55 SWE−FIS−54 SWE−FIS−64 SWE−FIS−PP66 SWE−FIS−63 SWE−FIS−35 SWE−FIS−53
SWE−BYN
SWE−BYN−4M SWE−BYN−8 SWE−BYN−1 SWE−BYN−10 SWE−BYN−11 SWE−BYN−5M SWE−BYN−17 SWE−BYN−18 SWE−BYN−19 SWE−BYN−15 SWE−BYN−2M SWE−BYN−3M SWE−BYN−12 SWE−BYN−16 SWE−BYN−1M SWE−BYN−PP54 SWE−BYN−PP81 SWE−BYN−82 SWE−BYN−90 SWE−BYN−PP78 SWE−BYN−PP65 SWE−BYN−94 SWE−BYN−PP42
SWE−GOT
SWE−GOT−10 SWE−GOT−4 SWE−GOT−15 SWE−GOT−9 SWE−GOT−17 SWE−GOT−3 SWE−GOT−7 SWE−GOT−8 SWE−GOT−20 SWE−GOT−16 SWE−GOT−2 SWE−GOT−5 SWE−GOT−1 SWE−GOT−13 SWE−GOT−12 SWE−GOT−14 SWE−GOT−6 SWE−GOT−11 SWE−GOT−19
1
0.8
0.6
0.4
0.2
0

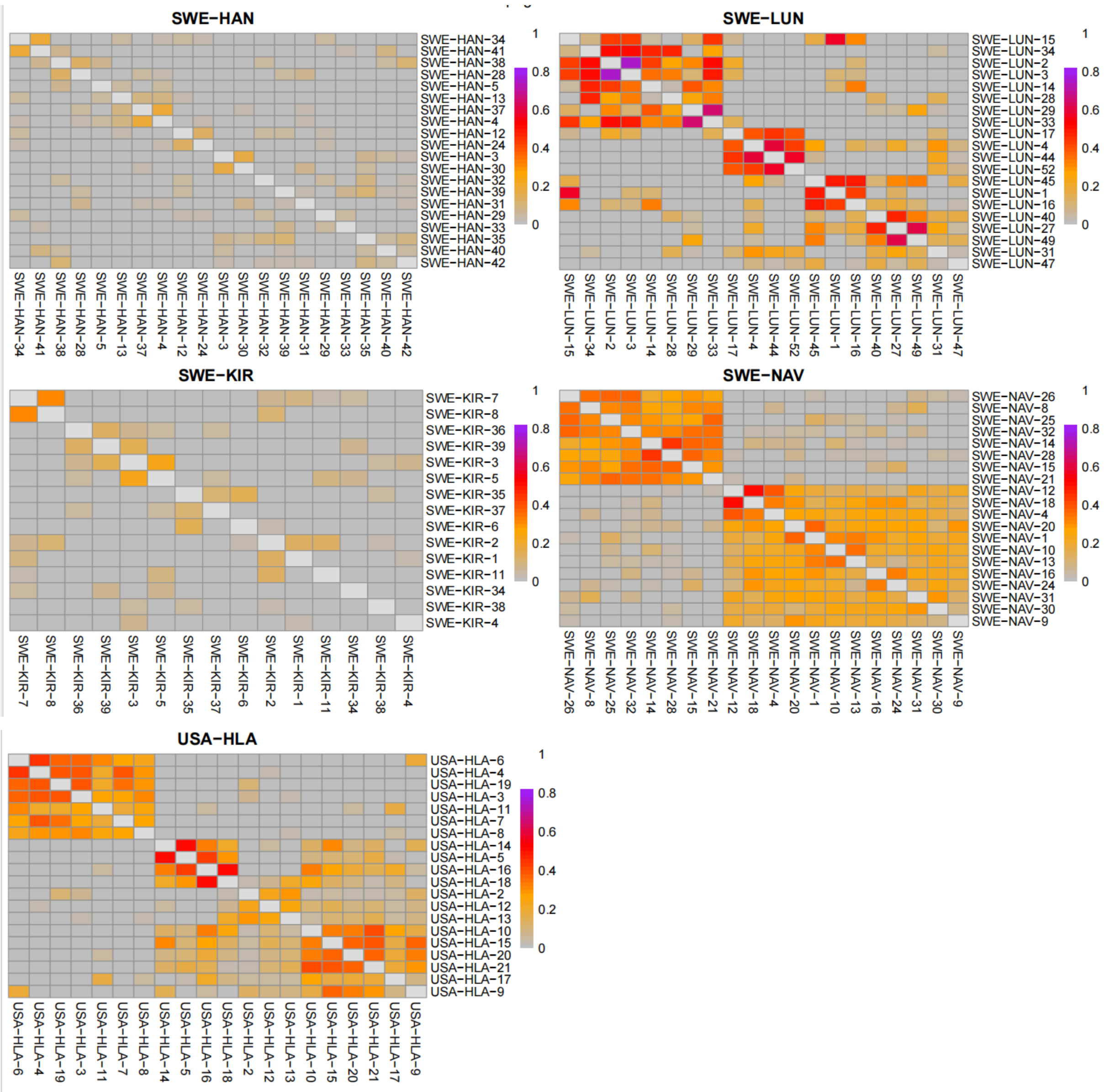

Figure S3. Relatedness (r_{xy}) for pairs of individuals within each population. Colors representing specific relatedness classes.

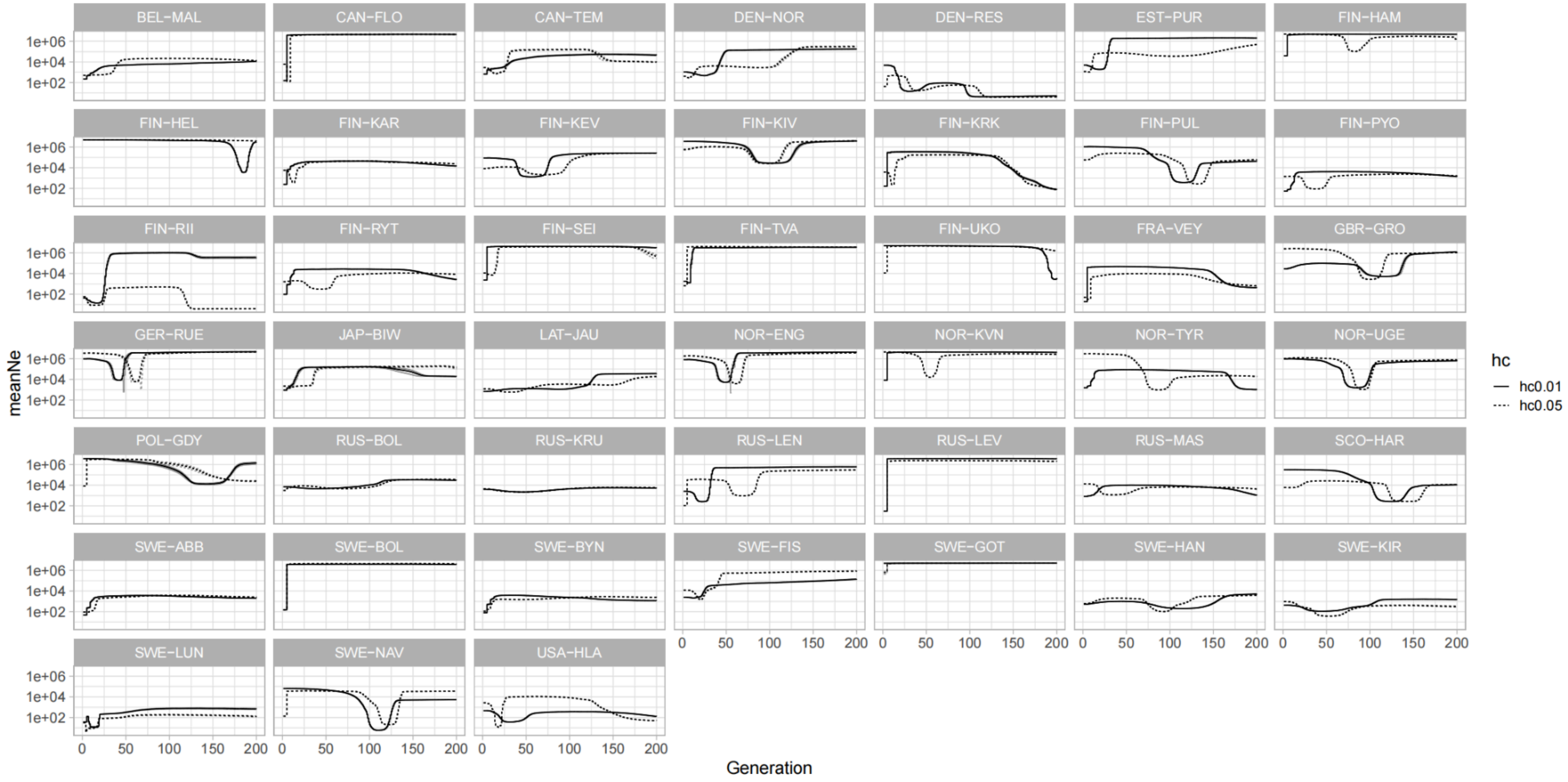

Figure S4. Effective population size (N_e) of each population estimated with GONE. The x-axis shows the time in generations before the sampling. Within each panel, the solid and dashed line correspond to the result of using a recombination fraction of 0.01 and 0.05, respectively.

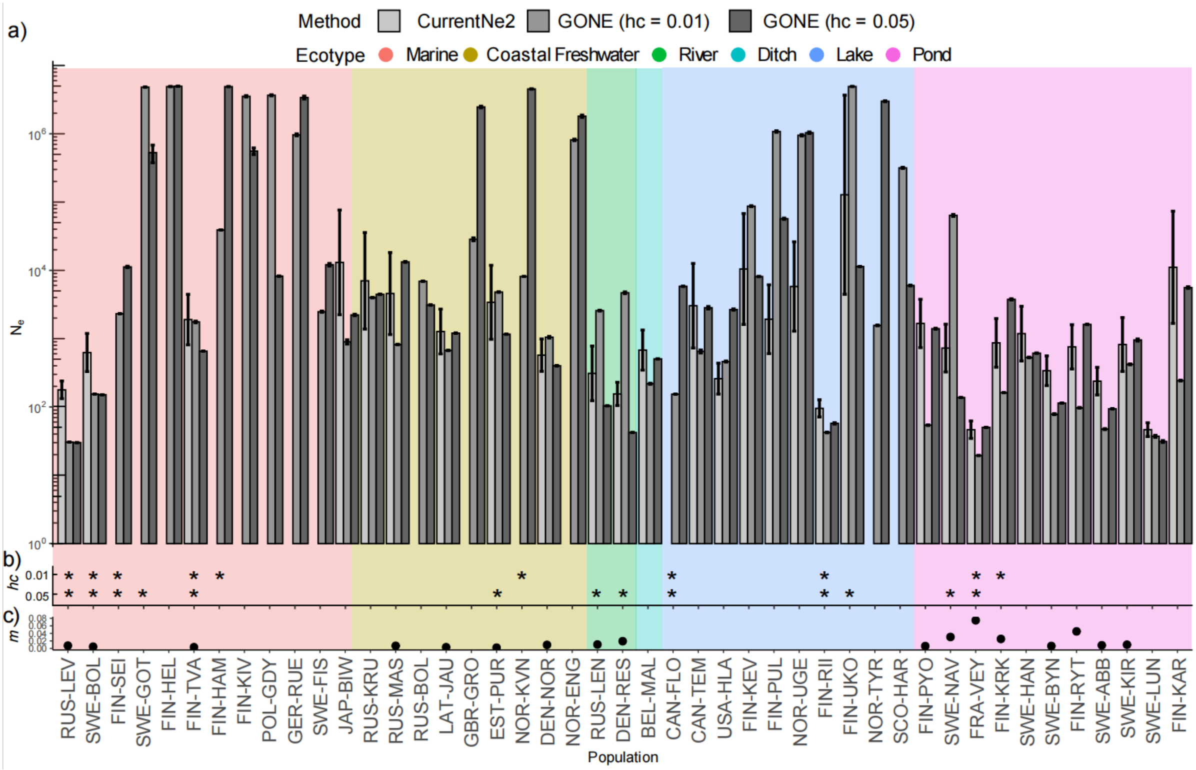

Figure S5. Estimates of contemporary effective population size (N_e^C) for 45 nine-spined stickleback populations. a). N_e^C and its 90% confidence intervals estimated using CurrentNe2 and GONE (with *hc* = 0.01 and 0.05). Populations are arranged in the same order as in Fig. 1 and the background color indicates the ecotype. b). Populations inferred to have experienced a drastic decline in N_e in GONE analysis. For exact N_e^C values, see Table S5.

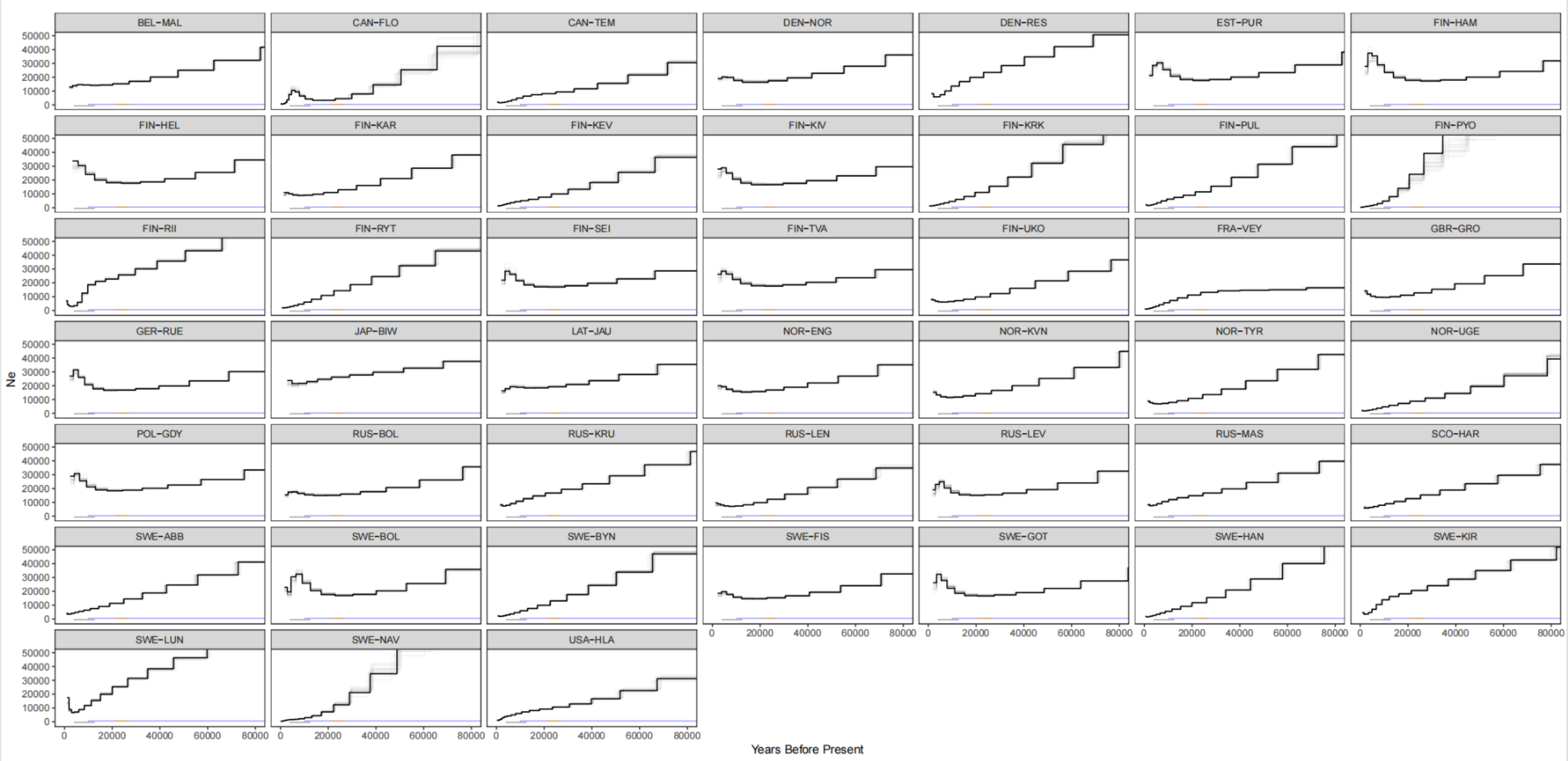

Figure S6. Historical effective population sizes (N_e) for each population inferred with MSMC2. The x-axis shows the time in years before present based on generation time of two years and mutation rate of 4.37×10^{-9} per base pair per generation. The grey, blue and orange bars at the bottom indicate the times for the formation of the Baltic Sea, the Last Glacial Period and the Last Glacial Maximum, respectively. Within each panel, the thin lines correspond to the original inferences and 20 rounds of bootstrap replicates, and the bold lines show the original inferences of each population.

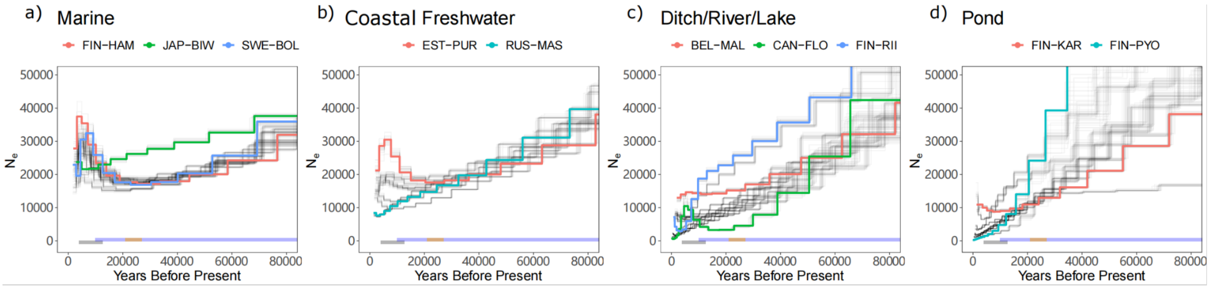

Figure S7. Historical effective population sizes (N_e) for populations within different ecotypes inferred with MSMC2. a) Marine populations. b) Coastal and freshwater populations. c) Lake and stream populations. d) Pond populations. The x-axis shows the time in years before present based on generation time of two years and mutation rate of 4.37×10-9 per base pair per generation. The grey, blue and orange bars at the bottom indicate the times for the formation of the Baltic Sea, the Last Glacial Period and the Last Glacial Maximum, respectively. Within each panel, the thin lines correspond to the original inferences and 20 rounds of bootstrap replicates, and the bold lines show representative populations from each ecotype.

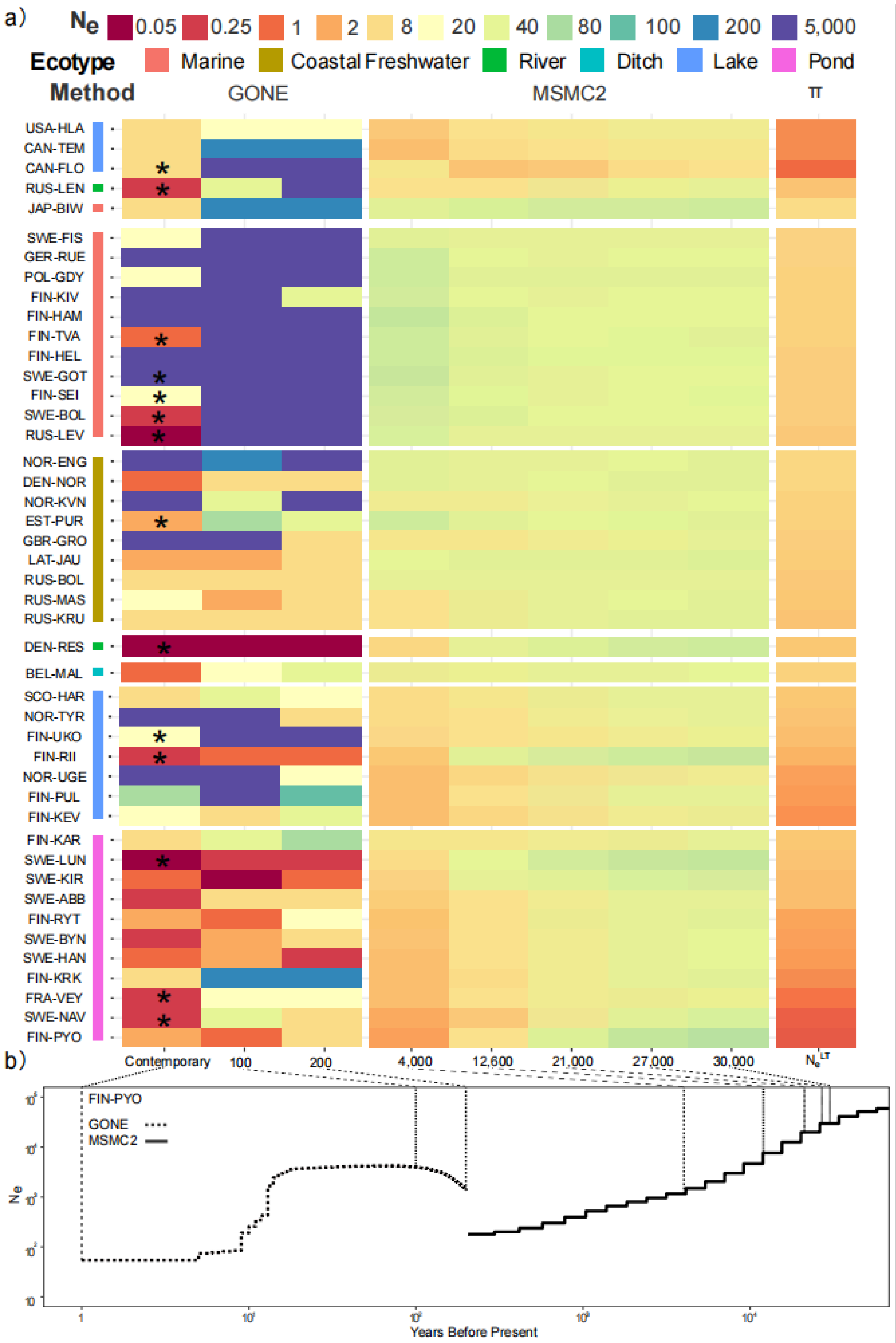

a)
Ne 0.05 0.25 1 2 8 20 40 80 100 200 5,000
Ecotype Marine Coastal Freshwater River Ditch Lake Pond
Method GONE MSMC2 π
USA-HLA
CAN-TEM
CAN-FLO
RUS-LEN
JAP-BIW
SWE-FIS
GER-RUE
POL-GDY
FIN-KIV
FIN-HAM
FIN-TVA
FIN-HEL
SWE-GOT
FIN-SEI
SWE-BOL
RUS-LEV
NOR-ENG
DEN-NOR
NOR-KVN
EST-PUR
GBR-GRO
LAT-JAU
RUS-BOL
RUS-MAS
RUS-KRU
DEN-RES
BEL-MAL
SCO-HAR
NOR-TYR
FIN-UKO
FIN-RII
NOR-UGE
FIN-PUL
FIN-KEV
FIN-KAR
SWE-LUN
SWE-KIR
SWE-ABB
FIN-RYT
SWE-BYN
SWE-HAN
FIN-KRK
FRA-VEY
SWE-NAV
FIN-PYO
Contemporary 100 200 4,000 12,600 21,000 27,000 30,000 Ne LT
b)
FIN-PYO
GONE
MSMC2
Ne
Years Before Present

Figure S8. Temporal reconstruction of effective population size (N_e) for 45 nine-spined stickleback populations over the past 30,000 years. a) The colors represent N_e estimates at key time points with the scale given at the top. The estimates for the last 200 years were derived with GONE (*hc* = 0.05), while those for 4,000 to 30,000 years ago were obtained with MSMC2. Asterisks indicate estimates influenced by population structure as inferred by manual inspection following the patterns in Fig. 2F of Santiago et al. (2020). Long-term N_e values were calculated from genetic diversity (π). Key time points were chosen to reflect significant geographic events: 4,000 years ago marks the onset of the current Baltic Sea stage, 12,600 years ago the start of the Baltic Ice Lake stage, and 21,000-27,000 years ago corresponds to the Last Glacial Maximum. The color next to population labels represents the ecotypes. b) The demographic history of a representative population, FIN-PYO, over the past 80,000 years. The dashed lines connect the time points in panels a) and b).

Tabel S1. Sample information.

Sample information							
Population	Latitude	Longitude	Habitat	Region	Number of individual	Sample year	Admixture Proportions (derived from Feng et al., (2022).)
BEL-MAL	51.17	3.47	Ditch	Central Belgium	20	2011	0.000
DEN-NOR	54.98	8.66	Coastal Freshwater	North Sea	25	2011	0.000
DEN-RES	56.18	9.63	River	Central Denmark	20	2015	0.000
EST-PUR	59.42	26.98	Coastal Freshwater	Coastal Baltic Sea	19	2006	0.000
FIN-HAM	60.56	27.19	Marine	Baltic Sea	20	2009	0.000
FIN-HEL	60.2	25.18	Marine	Baltic Sea	22	2007/2008/2010	0.119
FIN-KAR	66.66	26.44	Pond	Northern Finland	20	2009	0.122
FIN-KEV	69.75	27.02	Lake	Northern Finland	20	2009	0.127
FIN-KIV	65	25.47	Marine	Baltic Sea	19	2003	0.016
FIN-KRK	66.44	29.14	Pond	Northern Finland	20	2006	0.000
FIN-PUL	69.97	27.97	Lake	Northern Finland	17	2009	0.114
FIN-PYO	66.26	29.43	Pond	Northern Finland	31	2006-2008	0.000
FIN-RII	68.11	23.57	Lake	Northern Finland	23	2009	0.000
FIN-RYT	66.38	29.32	Pond	Northern Finland	21	2006-2008,2010	0.000
FIN-SEI	60.23	21.95	Marine	Baltic Sea	24	2010	0.000

FIN-TVA	59.83	23.2	Marine	Baltic Sea	22	2009	0.000
FIN-UKO	68.78	27.44	Lake	Northern Finland	22	2010	0.139
FRA-VEY	46.23	5.12	Pond	Central France	16	2009	0.134
GBR-GRO	57.62	-7.51	Coastal Freshwater	British Isles	19	2015	0.000
GER-RUE	54.01	13	Marine	Baltic Sea	27	2009	0.000
LAT-JAU	57.51	21.69	Coastal Freshwater	Coastal Baltic Sea	21	2009	0.000
NOR-ENG	59.9	10.53	Coastal Freshwater	Skagerrak	23	2008	0.343
NOR-KVN	70.58	26.99	Coastal Freshwater	Barents Sea coast	11	NA	0.000
NOR-TYR	59.91	10.3	Lake	Skagerrak	8	2006	0.162
NOR-UGE	63.96	10.43	Lake	Coastal Norwegian Sea	20	2006/2007	0.195
POL-GDY	54.4	18.53	Marine	Baltic Sea	20	2009	0.000
RUS-BOL	66.3	33.4	Coastal Freshwater	White Sea	20	2007	0.153
RUS-KRU	66.3	33.4	Coastal Freshwater	White Sea	20	2007	0.000
RUS-LEV	66.3	33.4	Marine	White Sea	30	2007	0.222
RUS-MAS	66.3	33.4	Coastal Freshwater	White Sea	21	2007	0.000
SCO-HAR	55.75	-4.42	Lake	British Isles	10	2007	0.000
SWE-ABB	64.48	19.44	Pond	Swedish	21	2003/20	0.000

				Inland		07	
SWE-BOL	63.66	20.21	Marine	Baltic Sea	21	2007	0.000
SWE-BYN	64.46	19.44	Pond	Swedish Inland	23	2003/2007	0.000
SWE-FIS	58.23	11.4	Marine	Kattegat	20	2009	0.000
SWE-GOT	57.73	18.95	Marine	Baltic Sea	19	2011	0.029
SWE-HAN	64.56	19.17	Pond	Swedish Inland	20	2007	0.124
SWE-KIR	67.9	20.09	Pond	Swedish Inland	15	2002	0.036
SWE-LUN	55.72	13.43	Pond	Baltic Sea (isolated)	21	2009	0.231
SWE-NAV	64.56	19.2	Pond	Swedish Inland	20	2007	0.136
CAN-FLO	54.23	-111.63	Lake	Canadian Inland	8	2011	0.025
CAN-TEM	47.71	-68.91	Lake	Canadian Inland	16	2010	0.023
JAP-BIW	43.08	145.11	Marine	Japan Sea	24	2007	0.341
RUS-LEN	72.98	126.96	River	Fareast	10	2014	0.037
USA-HLA	61.59	-149.76	Lake	Alaskan	20	2015	0.000

Table S2. Data filtering for each analysis.

Name	Filtering Criteria	Used in analysis	Number of SNPs
SNP Set 0	Original dataset, removing identified repetitive sequences and masked variants, only autosomal biallelic SNPs retained	NA	NA
SNP Set 1	From SNP Set 0, keep only binary SNPs with quality score ≥ 30, mean coverage ≥ 8x and ≤25x, GQ ≥ 20, allowing maximum 25% missing data	GONE	15,217,577
SNP Set 2	From SNP Set 1,keep only linkage group LG1 and LG4	PopLDdecay, ngsRelate, Inbreeding Coefficients	LG1: 1,124,554 LG4: 1,276,508
Bam Alignments Set 1	ANGSD Command "-uniqueOnly 1 -remove_bads 1 -skipTriallelic 1 -minMapQ 20 -minQ 20 -doHWE 1 -C 50" "-doMajorMinor 1 -doMaf 1 -dosnpstat 1 -doPost 2 -doGeno 11" "-doSaf 1 -fold 1 -anc $REF -ref $REF"	Genetic Diversity and Long-term Ne estimation	NA
Bam Alignments Set 2	https://github.com/stschiff/msmc-tools/tree/master	MSMC2	NA

Table S3. Number of SNPs used in GONE analysis.

Population	Number of SNPs	Population	Number of SNPs
BEL-MAL	1067280	NOR-TYR	733880
DEN-NOR	1341360	NOR-UGE	689760
DEN-RES	925600	POL-GDY	1475960
EST-PUR	1201680	RUS-BOL	990480
FIN-HAM	1170400	RUS-KRU	728760
FIN-HEL	1056120	RUS-LEV	814960
FIN-KAR	772920	RUS-MAS	668960
FIN-KEV	419120	SCO-HAR	1106200
FIN-KIV	1248680	SWE-ABB	482120
FIN-KRK	276040	SWE-BOL	1018600
FIN-PUL	422800	SWE-BYN	378560
FIN-PYO	85480	SWE-FIS	1277120
FIN-RII	486320	SWE-GOT	1239000
FIN-RYT	349400	SWE-HAN	356840
FIN-SEI	1299680	SWE-KIR	523040
FIN-TVA	1302720	SWE-LUN	829680
FIN-UKO	636040	SWE-NAV	322360
FRA-VEY	680360	CAN-FLO	940040
GBR-GRO	1270040	CAN-TEM	1473840
GER-RUE	1472800	JAP-BIW	1706440
LAT-JAU	1124440	RUS-LEN	436520
NOR-ENG	1499240	USA-HLA	457960
NOR-KVN	1148600		

Table S4. Number of SNPs used in CurrentNe2 analysis.

Population	Number of SNPs	Population	Number of SNPs
BEL-MAL	151250	NOR-TYR	649934
DEN-NOR	381298	NOR-UGE	132514
DEN-RES	945480	POL-GDY	419635
EST-PUR	63687	RUS-BOL	317369
FIN-HAM	112582	RUS-KRU	335403
FIN-HEL	230457	RUS-LEV	743222
FIN-KAR	104746	RUS-MAS	325058
FIN-KEV	76665	SCO-HAR	88352
FIN-KIV	93576	SWE-ABB	104502
FIN-KRK	159380	SWE-BOL	56594
FIN-PUL	412560	SWE-BYN	53606
FIN-PYO	33254	SWE-FIS	570739
FIN-RII	186410	SWE-GOT	39871
FIN-RYT	3960	SWE-HAN	86221
FIN-SEI	88599	SWE-KIR	39771
FIN-TVA	26215	SWE-LUN	174338
FIN-UKO	235386	SWE-NAV	481087
FRA-VEY	548460	CAN-FLO	32375
GBR-GRO	130426	CAN-TEM	242236
GER-RUE	557882	JAP-BIW	262119
LAT-JAU	170917	RUS-LEN	115353
NOR-ENG	90250	USA-HLA	65951
NOR-KVN	79716		

Table S5. Estimates of contemporary effective population size (NeC) using CurrentNe2 and GONE.

Population	GONE (hc=0.01)				GONE (hc=0.05)				CurrentNe2 (Default)			CurrentNe2 (Allow migration)				
	NeC	90% CI Lower	90% CI Upper	Affected by population structure	NeC	90% CI Lower	90% CI Upper	Affected by population structure	NeC	90% CI Lower	90% CI Upper	NeC	90% CI Lower	90% CI Upper	m (migra tion rate)	Affected by population structure
BEL-MAL	219	215	223	FALSE	507	506	509	FALSE	609	319	1162	682	347	1340	0.0107	TRUE
CAN-FLO	154	154	154	TRUE	5855	5824	5886	TRUE	NA	NA	NA	NA	NA	NA	NA	NA
CAN-TEM	652	619	685	FALSE	2847	2727	2968	FALSE	3045	732	12658	NA	NA	NA	NA	NA
DEN-NOR	1053	1025	1082	FALSE	402	398	406	FALSE	347	221	543	575	333	992	0.0095	TRUE
DEN-RES	4713	4573	4853	FALSE	42	42	42	FALSE	85	62	115	156	105	230	0.0193	TRUE
EST-PUR	4828	4784	4872	FALSE	1165	1160	1170	FALSE	477	263	866	3413	980	11886	0.0022	TRUE

FIN-HAM	39272	39230	39314	TRUE	4924781	4922319	4927243	FALSE	NA	NA	NA	NA	NA	NA	NA	NA
FIN-HEL	4931664	4929770	4933557	FALSE	4979979	4979155	4980802	FALSE	NA	NA	NA	NA	NA	NA	NA	NA
FIN-KAR	244	241	247	FALSE	5626	5470	5781	FALSE	11125	1679	73725	NA	NA	NA	NA	NA
FIN-KEV	87322	86002	88643	FALSE	8144	8105	8184	FALSE	10503	1619	68139	NA	NA	NA	NA	NA
FIN-KIV	3548821	3461886	3635756	FALSE	560489	498679	622298	FALSE	NA	NA	NA	NA	NA	NA	NA	NA
FIN-KRK	162	162	163	TRUE	3776	3699	3854	FALSE	843	375	1895	870	383	1974	0.0253	TRUE
FIN-PUL	1083652	1055711	1111592	FALSE	57217	55981	58453	FALSE	1931	606	6155	NA	NA	NA	NA	NA
FIN-PYO	54	54	55	FALSE	1403	1381	1426	FALSE	144	105	198	1674	741	3781	0.0063	TRUE
FIN-RII	42	42	43	TRUE	58	56	59	TRUE	96	72	128	NA	NA	NA	NA	NA

FIN-RYT	97	96	99	FALSE	1621	1611	1630	FALSE	796	372	1701	761	361	1606	0.0453	TRUE
FIN-SEI	2324	2320	2328	TRUE	11275	11002	11548	TRUE	NA	NA	NA	NA	NA	NA	NA	NA
FIN-TVA	1763	1714	1811	TRUE	658	657	658	TRUE	686	386	1222	1907	814	4469	0.0034	TRUE
FIN-UKO	4939132	4937442	4940823	FALSE	11428	11425	11432	TRUE	128559	4478	3690590	NA	NA	NA	NA	NA
FRA-VEY	20	20	20	TRUE	50	50	50	TRUE	53	39	72	47	35	62	0.0748	TRUE
GBR-GRO	28620	27222	30017	FALSE	2479610	2410518	2548703	FALSE	NA	NA	NA	NA	NA	NA	NA	NA
GER-RUE	969743	938261	1001226	FALSE	3409029	3242053	3576006	FALSE	NA	NA	NA	NA	NA	NA	NA	NA
JAP-BIW	900	837	964	FALSE	2233	2176	2289	FALSE	13125	2246	76732	NA	NA	NA	NA	NA
LAT-JAU	674	669	679	FALSE	1210	1200	1219	FALSE	269	178	407	1276	600	2714	0.0033	TRUE

NOR-ENG	816746	796981	836511	FALSE	1816540	1746073	1887007	FALSE	NA	NA	NA	NA	NA	NA	NA	NA
NOR-KVN	8151	8146	8155	TRUE	4545163	4516031	4574294	FALSE	NA	NA	NA	NA	NA	NA	NA	NA
NOR-TYR	1568	1549	1587	FALSE	3010800	2968553	3053048	FALSE	NA	NA	NA	NA	NA	NA	NA	NA
NOR-UGE	954640	932017	977263	FALSE	1040606	1013951	1067261	FALSE	5824	1292	26267	NA	NA	NA	NA	NA
POL-GDY	3677198	3613239	3741157	FALSE	8263	8201	8324	TRUE	NA	NA	NA	NA	NA	NA	NA	NA
RUS-BOL	6939	6896	6982	FALSE	3120	3096	3145	FALSE	NA	NA	NA	NA	NA	NA	NA	NA
RUS-KRU	4001	3963	4040	FALSE	4472	4444	4500	FALSE	7050	1386	35882	NA	NA	NA	NA	NA
RUS-LEN	2582	2545	2620	FALSE	104	104	104	TRUE	63	38	104	311	124	781	0.0105	TRUE
RUS-LEV	31	31	31	TRUE	30	30	30	TRUE	11	10	12	179	133	240	0.0075	TRUE

RUS-MAS	823	814	832	FALSE	13347	13157	13537	FALSE	3160	943	10589	4591	1152	18299	0.0070	TRUE
SCO-HAR	318071	311519	324623	FALSE	6020	5946	6094	FALSE	NA	NA	NA	NA	NA	NA	NA	NA
SWE-ABB	48	47	48	FALSE	94	94	95	FALSE	165	110	247	239	150	381	0.0085	TRUE
SWE-BOL	155	155	155	TRUE	151	151	151	TRUE	417	241	722	628	331	1195	0.0047	TRUE
SWE-BYN	79	77	80	FALSE	114	114	115	FALSE	63	49	82	340	206	561	0.0066	TRUE
SWE-FIS	2489	2438	2541	FALSE	12137	11610	12665	FALSE	NA	NA	NA	NA	NA	NA	NA	NA
SWE-GOT	4835839	4815328	4856350	FALSE	531713	378211	685215	FALSE	NA	NA	NA	NA	NA	NA	NA	NA
SWE-HAN	532	526	538	FALSE	615	611	620	FALSE	1188	473	2984	NA	NA	NA	NA	NA
SWE-KIR	422	416	429	FALSE	959	926	992	FALSE	278	153	507	824	332	2042	0.0100	TRUE

SWE-LUN	37	36	39	FALSE	31	30	32	FALSE	47	37	59	NA	NA	NA	NA	NA
SWE-NAV	63944	61892	65995	FALSE	138	137	138	TRUE	245	144	416	730	326	1631	0.0305	TRUE
USA-HLA	464	456	472	FALSE	2678	2621	2735	FALSE	260	155	438	NA	NA	NA	NA	NA
